# Association of preoperative ultrasonographic parameters of the contralateral kidney with long-term serum creatinine in cats treated for unilateral ureteral obstruction

**DOI:** 10.3389/fvets.2025.1518713

**Published:** 2025-01-22

**Authors:** Diego Pulido Vega, Jérémie Ficheroulle, Mathieu Manassero, Jeremy Mortier, Christelle Maurey

**Affiliations:** ^1^Service of Diagnostic Imaging, DEPEC, École Nationale Vétérinaire d’Alfort, Maisons-Alfort, France; ^2^Teaching and Clinical Department of Companion Animal, Faculty of Veterinary Medicine, Fundamental and Applied Research for Animals and Health, University of Liège, Liège, Belgium; ^3^Service of Small Animal Surgery, École Nationale Vétérinaire d’Alfort, Maisons-Alfort, France; ^4^Laboratoire de Santé Animale, Service of Internal Medicine, École Nationale Vétérinaire d’Alfort, INRAE, ANSES, UMR Virology, Maisons-Alfort, France

**Keywords:** sub, ureterolithiasis, ureteral calculi, kidney, ureter

## Abstract

**Introduction:**

Prediction of renal recovery after surgical management of feline unilateral ureteral obstruction (UO) is crucial to guide therapeutic decisions, but predictors of this outcome are still lacking. Despite the functional importance of the contralateral kidney, there is currently no precise description of its ultrasonographic (US) features. In addition, US parameters of both the renal collecting system and the renal parenchyma have been identified in human medicine as prognostic factors in the case of UO but have not been described in veterinary medicine. The aim of this study was to evaluate an association between preoperative structural US renal parameters and long-term International Renal Interest Society (IRIS) stage after successful renal decompression with subcutaneous ureteral bypass (SUB) device in cats with unilateral UO.

**Methods:**

This retrospective study included 60 cats with unilateral UO and evaluated preoperative US parameters of both kidneys, including measurements of parenchymal and pelvic areas as well as a renal score. Cats were divided according to their serum creatinine at 3 months postoperatively into group A (IRIS stages I and II) and group B (IRIS stages III and IV).

**Results:**

A higher US chronic kidney disease (US-CKD) score of the kidney contralateral to the UO was associated with long-term IRIS stages III and IV. It also appeared as a fair discriminator of long-term IRIS stage IV, with an area under the curve of 0.74. The optimal cutoff value for accurately identifying cats with long-term IRIS stage IV was a US-CKD score > 7, with a specificity of 98%, a sensitivity of 25%, and a positive likelihood ratio of 12.75. No preoperative US parameters regarding the obstructed kidney, including parenchymal and pelvic areas, were significantly associated with long-term creatinine.

**Conclusion:**

Ultrasonographic scoring of contralateral chronic kidney disease abnormalities is associated with IRIS stage following treatment of feline unilateral UO with a SUB device and serves as a specific indicator of cats presenting with long-term IRIS stage IV.

## 1 Introduction

Ureteral obstruction (UO) is a common cause of acute azotemia in cats, frequently occurring concurrently with chronic kidney disease (CKD) (1–3). Its most common etiology is ureterolithiasis, which is predominantly composed of calcium oxalate (4–11). Treatment of UO is challenging, and a subcutaneous ureteral bypass (SUB) device placement is considered an effective therapeutic strategy, with a low postoperative death rate and an excellent outcome (3, 12). However, this technique comes with high costs for the owners, a demanding follow-up, and a significant rate of long-term complications. In addition, postoperative renal function often remains impaired after the acute episode, reflecting not only the effects of concurrent CKD but also the sequelae of UO, with half of cats having a serum creatinine (SCr) greater than 3.2 mg/dL 6 months after surgery (3, 12–14).

In this context, the identification of prognostic factors is crucial to assist clinicians and pet owners in the surgical decision-making process. In two previous studies (9, 15), no preoperative clinical, biochemical, or ultrasonographic (US) finding was found predictive of long-term renal recovery, although the focus was on the imaging characteristics of the obstructed kidney alone. The McEntee et al. study (15) introduced a ratio of pelvic dilation to overall renal size which was not predictive of long-term SCr. Similar parameters assessing areas, rather than lengths, of both the renal parenchyma and the pelvic dilation have been described in human medicine. For instance, the parenchyma-to-hydronephrosis area ratio (PHAR) has proved to be predictive of renal function recovery after surgery in the context of ureteropelvic junction obstruction (16, 17), but it has not been studied in the context of UO in veterinary medicine.

To the best of the author’s knowledge, the US description of feline unilateral UO only focuses on the obstructed kidney, with no mention other than the size of the contralateral kidney (15, 18–22). Given that azotemia remains undetected until 75% of the renal function has been lost (23), unilateral UO does not result in azotemia if the contralateral kidney is functioning well. However, in cats, UO occurs in the course of a CKD, possibly fostered through repeated asymptomatic ureteral obstructive episodes (5), contributing to pre-existing renal damage within the contralateral kidney and explaining why UO becomes clinically relevant (24). This pathophysiological sequence underlines the need for a careful assessment of the contralateral kidney.

The aims of this study were (1) to assess the replicability of the research of McEntee et al. (15) performed on the obstructed kidney with a focus on additional novel US parameters and (2) to extend their work by evaluating ultrasonographically the contralateral kidney with the hypothesis that some parameters would show an association with the long-term renal recovery after pelvic decompression with a SUB device.

## 2 Materials and methods

### 2.1 Selection of cases

Medical and US records of all cats treated with a SUB device (Norfolk Vet Products) between December 2013 and January 2021 at the National Veterinary School of Alfort were retrospectively reviewed. Cats were included in the study if they presented a benign unilateral UO, renal preoperative US images and were followed for at least 3 months postoperatively with ultrasonographic evidence of renal pelvic decompression and patency of the device. Cats that died from renal causes during the 3-month follow-up were also included. Ureteral obstruction was diagnosed based on clinical signs, the presence of azotemia, and US findings such as pelvic dilation, diverticular dilation, ureteral dilation, intraluminal obstructive lesion, or some combination of these. Cats were treated in accordance with the standard surgical and perioperative management protocols, as previously described (3). Long-term follow-up was determined as the first follow-up occurring more than 3 months postoperatively. Additional data were collected and reviewed on a case-by-case basis, by a board-certified veterinary specialist in internal medicine (CM), to identify cats presenting with an acute or chronic kidney disease at the time of the long-term follow-up. This was based on a combination of criteria such as an increase in SCr higher than 20% above the last SCr measurement, acute onset of clinical signs (e.g., anorexia, lethargy, and vomiting), US images at the time of long-term follow-up compared with previous examinations, urine culture, and serum amyloid A protein (SAA) when available. For these cats, the long-term follow-up date was determined as the first follow-up in which the acute or chronic kidney event was resolved. Cats with bilateral UO, ureteral rupture, and UO of neoplastic origin, cats treated as part of a second surgery for UO, with incomplete medical records, lost to follow-up, or deceased from unknown or non-renal cause before long-term follow-up were excluded.

### 2.2 Clinical and biological data

Preoperative standardized clinical data were gathered from all cats including age, sex, breed, weight, nature, and duration of clinical signs. Blood samples and chemistry were collected. During surgery, pelvic urine from the obstructed kidney was collected for bacterial culture. Postoperative data included SCr follow-up for at least 3 months.

### 2.3 Ultrasonographic findings

**Figure 1:**
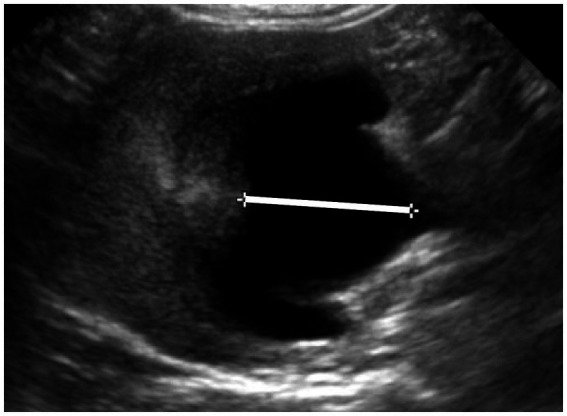
Ultrasonographic image of an obstructed kidney, in a transverse plane. The white line indicates the pelvic dilatation, spanning from one edge of the pelvis to the other, with a width of 7 mm.

**Figure 2:**
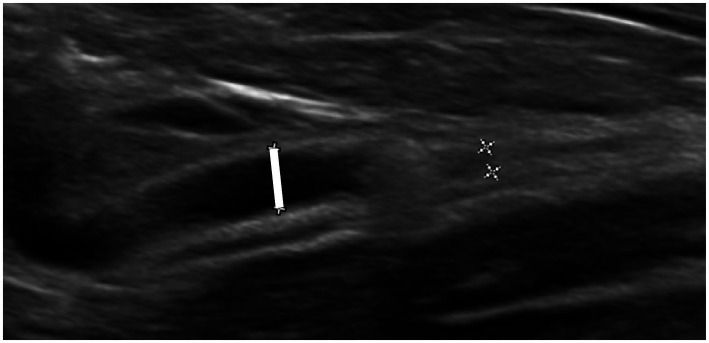
Ultrasonographic image of a ureter with intraluminal calculi. The ureter is dilated upstream of the calculi, and its diameter is indicated with the white line (measuring 2.3 mm), with no downstream dilation.

**Figure 3:**
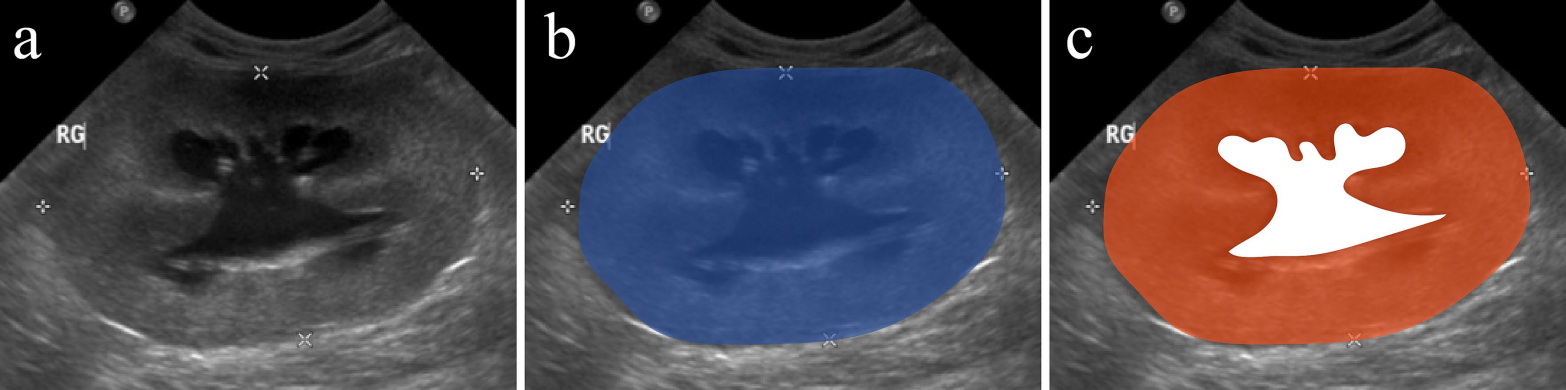
Parenchyma-to-hydronephrosis area ratio (PHAR) measurement on an ultrasonographic image of a kidney with hydronephrosis. Sagittal image of a hydronephrotic kidney **(A)**. The blue area **(B)** corresponds to the total renal area. The white area **(C)** corresponds to the pelvic area. The renal parenchymal area (RPA) was calculated by subtracting the pelvic area from the total renal area and corresponds to the orange area **(C)**. The PHAR was calculated by dividing the RPA by the pelvic area.

The US examinations were performed using different ultrasound machines over the study time (IU22, Philips; Affinity 50, Philips) and by various experienced ultrasonographers. A multifrequency (5–8 MHz) microconvex or a multifrequency (5–18 MHz) linear transducer was used for ultrasound examination. Preoperative abdominal US still images were blindly reviewed by a veterinary radiology resident (JF). Measurements were performed for each kidney using the National Institutes of Health image software (Image J 1.53 k). Renal length was determined as the maximum longitudinal dimension in a sagittal plane. Pelvic diameter was measured in sagittal and transverse planes from one pelvic margin to another (Figure 1). Renal parenchymal thickness was defined as the distance between the renal sinus fat to the renal capsule in a sagittal plane. Renal cortical thickness was determined in a sagittal plane as the distance from the corticomedullary interface to the renal capsule. Cranial ureteral diameter was measured just caudally to the ureteropelvic junction, in the transverse plane. Ureteral diameter upstream of the obstruction site was measured in the sagittal plane, if an obstruction site was identified (Figure 2). The total renal area and pelvic area were determined by outlining the kidney and the pelvis on a sagittal plane as previously described (Figure 3) (25–28). The renal parenchymal area (RPA) was calculated by subtracting the pelvic area from the total renal area. Total RPA was defined as the sum of the RPA of both kidneys. Parenchyma-to-hydronephrosis area ratio (PHAR) was calculated by dividing the RPA by the pelvic area, only for obstructed kidneys.

To grade the severity of the CKD on US images, we proposed a US-CKD score. This score allowed a semi-quantitative US description of the kidneys based on the presence of findings reportedly associated with CKD including corticomedullary differentiation attenuation, medullary rim sign, irregular renal margination, renal infarcts, and renal cysts (29–32). A sub-score was assigned to each US finding according to their presence or severity as described in Table 1. The ultrasonographic CKD score was defined as the sum of each sub-score for one kidney (ranging from 0 to 8), and the total US-CKD score was defined as the sum of the US-CKD score of both kidneys.

**Table 1:** Ultrasonographic characteristics assessed to calculate the ultrasonographic chronic kidney disease score (US-CKD score).

Parameter	Sub-score	
Corticomedullary differentiation	0	Good
	1	Poor
	2	Absent
Medullary rim sign	0	Absent or ⩽ 2 mm
	1	Present and > 2 mm
Irregular renal margination	0	Absent
	1	Mild
	2	Moderate
	3	Marked
Renal infarcts	0	Absent
	1	Present
Renal cysts	0	Absent
	1	Present

### 2.4 Groups

Cats were divided into two groups based on their long-term renal recovery. Group A included cats with SCr ⩽ 2.8 mg/dL, corresponding to stages I and II of the International Renal Interest Society (IRIS) classification (33). Group B included cats with SCr > 2.8 mg/dL and cats that died from renal causes during the 3-month follow-up period. The SCr cutoff was based on survival analysis of existing data: Median survival time was superior to 2,250 days in stage IRIS stages I and II cats, while it was 608 and 67 days in IRIS stages III and IV cats, respectively, based on their SCr measured 3 months postoperatively in one study (3).

### 2.5 Data analysis and statistics

Statistical analysis was performed using a statistical package (BiostaTGV, Pierre Louis Institute of Epidemiology and Public Health UMR S 1136). Quantitative descriptive results are presented as medians with ranges. Dichotomous variables are presented as percentages and associated with the number of cases included. Association between US features and groups was assessed using the Mann–Whitney U-test for continuous data and either the χ^2^ test or Fisher’s exact test for categorical data. The Wilcoxon signed-rank test was used for comparison between the change in SCr between the preoperative period and long-term follow-up. Receiver operating characteristic (ROC) curves were used to assess the diagnostic utility of quantitative US parameters that differed significantly between groups. The generalized Youden index was used to identify optimal cutoff values. An AUC of >0.9 was interpreted as excellent, 0.8 to 0.89 as good, 0.7 to 0.79 as fair, 0.6 to 0.69 as poor, and 0.5 to 0.59 as failure (34), and values are accompanied by their respective 95% confidence intervals (CIs). The level of statistical significance selected was set at a *p*-value of <0.05.

## 3 Results

### 3.1 Study population

Ninety-eight cats were treated with an SUB device between December 2013 and January 2021 and recruited for the study. Thirty-eight cats were excluded as follows: 25 due to admission criteria and 13 due to follow-up criteria (see Supplementary Figure S1). Fifteen cats presented a bilateral UO, six cats presented a ureteral rupture, and four cats were treated as part of a second surgery on UO. During the follow-up period, one cat presented a contralateral UO, two cats presented an obstruction of the SUB device, and four cats were lost to follow-up. One cat was excluded because of truncated US images. Three cats died of unknown reasons after discharge, and two cats died from non-renal causes: One presented with respiratory distress and signs of congestive heart failure, and the second presented a resolution of azotemia after treatment but died after a blood transfusion. The remaining 60 cats were included in the study, of which four cats died or were euthanized due to persistent marked azotemia and did not survive discharge (range 2–8 days postoperatively). These cats were part of group B, along with cats presenting a long-term SCr > 2.8 mg/dL.

### 3.2 Clinical data

Descriptive statistics on the study population are summarized in Table 2. Of the 60 cats, 38 (63%) were females and 22/60 (37%) were males, of which 31/38 (82%) and 19/22 (86%) were neutered. The median age at presentation was 6.1 years (range 1.5–15.7 years). On admission, median packed cell volume (PCV) was 30% (range 17–45%), and median SCr was 5.9 mg/dL (range 1.4–18.1) with 59/60 (98%) cats being azotemic. Other biochemical abnormalities included anemia (42%, *n* = 25/59), hyperphosphatemia (36%, *n* = 9/25), hyperkalemia (20%, *n* = 12/59), and ionized hypercalcemia (10%, *n* = 5/50). Urine culture obtained by pyelocentesis at the time of SUB placement was positive in 11/59 (18.6%) cats. The median long-term follow-up was 4 months (range 3–8 months). The median long-term SCr was 2.3 mg/dL (range 1.2–5.4 mg/dL), which was significantly lower than preoperative values (*p* < 0.001) and associated with a median decrease in SCr of 3 mg/dL (range 0.1–16.4 mg/dL). Among the 60 cats included, 43 (72%) presented a long-term SCr ⩽ 2.8 mg/dL and were part of group A. The remaining 17 cats (28%) were part of group B and included 12 cats with long-term SCr > 2.8 mg/dL and 5 cats that died of renal causes, as described above. Eleven cats presented bacteriuria on urine collected from the SUB device at the time of long-term follow-up. Of these cats, one presented an acute or chronic kidney disease suspected to be pyelonephritis, based on the elevation of SCr, bacteriuria, evocative US findings, and increased SAA. In this case, the long-term follow-up date was set 1.5 months later, when the acute or chronic kidney event had resolved (SCr = 2.5 mg/dL and normalization of SAA). The remaining cats were asymptomatic, and none were suspected of presenting an acute kidney injury at this time based on clinical signs, biological data, and evaluation of serial postoperative SCr values at follow-up. Female cats were significantly more frequent in group A than in group B (72% vs. 41%; *p* = 0.03). The remaining clinical data were not significantly different between groups (Supplementary Table S1).

**Table 2:** Descriptive statistics in cats with unilateral ureteral obstruction treated with a SUB device.

	Median	Inter-quartile range	Number of cats	Reference interval
Age (years)	6.1	4.5–8	60	
Weight (kg)	3.6	3.3–4.4	60	
Duration of clinical signs (days)	10	7–23	53	
Total follow-up duration after surgery (years)	1.4	0.7–2.6	60	
Preoperative PCV (%)	30	25.5–32.2	59	29–48
Preoperative creatinine (mg/dL)	5.9	3.4–9.1	60	0.52–1.78
Preoperative phosphorus (mmol/L)	69	55–97	25	32–78
Preoperative ionized calcium (mmol/L)	1.27	1.20–1.33	50	1.10–1.40
Preoperative potassium (mmol/L)	4.6	4.0–5.4	59	3.6–5.5
Long-term creatinine (mg/dL)	2.3	1.8–2.7	56	0.52–1.78
Long-term follow-up duration (months)	4	3–5	56	

SUB = subcutaneous ureteral bypass; PCV = packed cell volume.

### 3.3 Ultrasonographic findings

Preoperative US data are summarized in Tables 3, 4. Thirty-five cats were obstructed on the right side (58%) and 25 on the left side (42%). Obstructed kidneys had a median length of 4.4 cm (range 2.3–6 cm) and presented a median pelvic diameter in the transverse plane of 7.2 mm (range 1.4–20.1 mm). Ipsilateral nephroliths were identified in 35 (58%) cats. Peri-renal effusion, peri-renal fat hyperechogenicity, attenuation of the corticomedullary distinction, and renal infarcts were identified in 10 (17%), 14 (23%), 40 (67%), and 32 (53%) cats, respectively. The median US-CKD score was 3 (range 0–7). Median cranial ureteral diameter and upstream obstruction site were 4.8 mm (range 1.5–14 mm) and 3 mm (range 1.6–19.3 mm), respectively. The contralateral kidney had a median length of 3.1 cm (range 1.2–4.9 cm). The most frequent structural abnormalities of this kidney were attenuation of the corticomedullary distinction, renal infarcts, moderately to markedly irregular margination, and pelvic mineralization in 53 (90%), 49 (83%), 44 (75%), and 40 (68%) cats, respectively. The median US-CKD score was 5 (range 0–8) which was significantly higher than in the obstructed kidney (*p* < 0.01; *n* = 59). Nephroliths were identified in 40 (68%) contralateral kidneys.

**Table 3:** Ultrasonographic parameters of the obstructed kidney in cats with unilateral ureteral obstruction.

	Median	Inter-quartile range	Number of cats
Renal length (cm)	4.4	3.9–4.6	60
Pelvic diameter in transverse plane (mm)	7.2	4.9–9.8	59
Pelvic diameter in sagittal plane (mm)	10.1	8.7–12.6	58
Renal cortical thickness (mm)	4.2	3.6–5	49
Renal parenchymal thickness (mm)	8.3	7–10.2	60
Cranial ureteral diameter (mm)	4.8	3.4–6.4	59
Ureteral diameter upstream obstruction site (mm)	3	2.2–3.6	52
RPA (cm^2^)	7.66	4.74–8.99	60
PHAR (cm^2^)	3.65	1.9–5.59	58
US-CKD score	3	2–5	60

RPA = renal parenchymal area; PHAR: parenchyma-to-hydronephrosis area ratio; US-CKD = ultrasonographic chronic kidney disease.

**Table 4:** Ultrasonographic parameters of the kidney contralateral to the ureteral obstruction in cats with unilateral ureteral obstruction.

	Median	Inter-quartile range	Number of cats
Renal length (cm)	3.1	2.5–3.6	59
Renal cortical thickness (mm)	3.4	2.9–4.1	46
Renal parenchymal thickness (mm)	7.4	5.7–8.8	58
RPA (cm^2^)	4.73	3.29–6.13	59
US-CKD score	5	4–6	59

RPA = renal parenchymal area; US-CKD = ultrasonographic chronic kidney disease.

The median US-CKD score of the contralateral kidney was significantly higher in group B (median, 5 in group A; median, 6 in group B; *p* = 0.01). Through ROC curve analysis, the US-CKD score of the contralateral kidney was a fair discriminator between groups A and B (AUC = 0.71, *p* < 0.01, 95% CI: 0.57–0.85). The optimal cutoff to correctly identify group B was a US-CKD score > 6 (specificity: 90.5%; sensitivity: 29.4%) and was associated with a likelihood ratio of 3.1 (95% CI: 0.94–4.94). Higher US-CKD score of the contralateral kidney was also significantly associated with cats presenting long-term CKD IRIS stage IV disease (median, 5 in IRIS stages I–III; median, 6 in stage IV; *p* = 0.028). The US-CKD score of the contralateral kidney was still a fair discriminator to distinguish cats presenting long-term CKD IRIS stage IV disease from others (AUC = 0.74; *p* = 0.02, 95% CI: 0.54–0.94). The optimal cutoff to correctly identify cats presenting long-term CKD IRIS stage IV disease was a US-CKD score > 7 (specificity: 98%; sensitivity: 25%) and was associated with a likelihood ratio of 12.75 (95% CI: 1.30–124.88). US-CKD score of the obstructed kidney was not significantly different among groups (median, 3 in each group; *p* = 0.56). Median total US-CKD score was higher in group B than in group A, but no statistical significance was reached (median, 8 in group A; median, 9 in group B; *p* = 0.08). No other preoperative US parameter, including RPA and PHAR, was significantly associated with long-term SCr (Table 5; Supplementary Table S2).

**Table 5:** Evaluation of the association between long-term serum creatinine and ultrasonographic parameters of the kidney contralateral to ureteral obstruction in included cats.

	Group A	Group B	*P*-value
Total renal area (cm^2^)	5 [3.8–6.7]	3.9 [3.3–6.7]	0.31
Renal parenchymal area: RPA (cm^2^)	5.0 [3.6–6.1]	3.7 [3.3–5.2]	0.17
Renal length (cm)	3.2 [2.5–3.6]	2.8 [2.5–3.6]	0.75
Renal cortical thickness (mm)	3.4 [2.9–4.2]	3.4 [3.1–3.7]	0.81
Renal parenchymal thickness (mm)	7.8 [5.8–8.9]	6.7 [4.7–7.7]	0.05
US-CKD score	5 [3–6]	6 [5–7]	0.01

Values are medians, and values in brackets represent the interquartile range. US-CKD = ultrasonographic chronic kidney disease.

## 4 Discussion

This retrospective study is the first to focus on the preoperative US appearance of the contralateral kidney to predict renal function recovery after surgical treatment of feline unilateral ureteral obstruction. A higher US-CKD score of the contralateral kidney was significantly associated with a poor outcome (SCr > 2.8 mg/dL) at 3 months postoperatively. A cutoff score of >6 discriminated these cats with a specificity of 90.5% and a sensitivity of 29.4%. These results suggest that the potential for renal recovery after unilateral SUB device placement should be discussed with owners in light of the US aspect of the contralateral kidney.

Despite its importance, the ultrasonographic description of the kidney contralateral to unilateral UO is scarce (15, 18–21) and limited to its size, reported as a mean of 6 mm smaller than the obstructed kidney (22). This phenomenon is referred to as “big kidney, little kidney” and results from repeated subclinical obstructive episodes leading to nephron loss and fibrosis (5, 19). To further characterize the contralateral kidney and to grade the US abnormalities associated with CKD, a US-CKD score was proposed. This score is based on the semi-quantitative evaluation of US structural indicators that are reportedly associated with CKD (28–32). In the present study, a higher preoperative contralateral US-CKD score was associated with long-term IRIS stages III and IV (group B). This is likely to have clinical implications as the latter are associated with median survival times of 608 days and 67 days, respectively, compared to more than 2,250 days in stage IRIS stages I and II, based on SCr measured 3 months postoperatively in one study (3). However, there is still a significant difference in median survival time within cats in group B. Therefore, we decided to identify a specific cutoff value to discriminate cats with long-term IRIS stage IV from others, and the value of US-CKD >7 showed a specificity of 98% and a sensitivity of 25%. This cutoff was also associated with an excellent likelihood ratio of 12.75 (95% CI: 1.30–124.88). Assuming that the pre-test probability of reaching IRIS stage IV in the long-term is similar to its prevalence in our study population (15%), this means that identifying preoperatively a US-CKD >7 in the contralateral kidney would substantially raise its post-test probability from 15 to 69% (35). It should be noted that this cutoff value is not sensitive and is primarily intended to identify cats that are at a high risk of poor renal recovery following surgery. Further research is required to validate prospectively the use of the US-CKD score in alternative contexts and more specifically to correlate its value with basal SCr values. Among other US parameters studied, the median parenchymal thickness of the contralateral kidney was lower in cats of group B (7.8 mm in group A; 6.7 mm in group B) even though this parameter did not reach statistical significance (*p* = 0.05). This is in accordance with a previous study that reported a decrease in renal cortical thickness in cats with impaired renal function (36). These findings highlight the importance of the contralateral kidney in the overall renal function following surgical intervention and advocate for its preoperative US assessment to aid in the surgical decision-making process.

In human medicine, area measurements have been developed to provide a more accurate and objective assessment of hydronephrotic kidneys with ultrasonography. The renal parenchymal area is highly and positively correlated with the renal volume measured by magnetic resonance imaging, with no effect of hydronephrosis on this correlation (37, 38) and serves as an early marker to predict future renal deterioration in infants with posterior urethral valves (39). The parenchyma-to-hydronephrosis area ratio is another parameter that combines the value of the parenchymal area with an objective measure of hydronephrosis. It is used to identify patients who are more suitable for surgery in the context of ureteropelvic junction obstruction as it has been found to predict postoperative renal function in humans undergoing pyeloplasty (16, 17, 38). In this study, it was hypothesized that these preoperative US structural parameters would correlate with nephron mass and serve as surrogate markers of functional renal reserve in cats with ureteral obstruction. However, parenchymal and hydronephrosis area measurements did not allow to predict renal recovery in this study, and one explanation could be that feline UO occurs in the context of CKD, in contrast to humans. Similarly, other regularly measured US parameters of the obstructed kidney did not correlate with long-term SCr, which aligns with the previous results of a recent study (15). These data support the argument that identification of US features such as severe hydronephrosis or thin parenchyma on the obstructed kidney should not deter surgical decompression in cats presenting with unilateral UO as there is no evidence to suggest that these features adversely impact long-term renal recovery.

Our study has a number of limitations. The sample size is moderate owing to the exclusion of a large number of cases, and this might have underpowered the statistical analyses. The long-term follow-up was set at a minimum of 3 months, and a longer period could have helped to better characterize renal recovery. This may only have little impact as other studies suggested overall stability of SCr after 3 months postoperatively (9, 12, 40). As acute events on CKD are part of this disease and may impact long-term outcomes, such cases were included in the study to avoid selection bias. This happened for one case of suspected pyelonephritis, and the long-term follow-up SCr was set when the acute event had resolved. Bacteriuria was detected in 11 cats at the date of follow-up, including the case of suspected pyelonephritis. The remaining cases presenting bacteriuria showed no clinical signs and an overall stability of SCr concentration at the date of long-term follow-up. A previous study reported that postoperative asymptomatic bacteriuria was not associated with survival and that no increase of SCr was observed in these cats (12). In our population, there was no significant difference in the proportion of cats presenting with long-term bacteriuria between the two groups (*p* = 1; *n* = 48). Owing to the retrospective nature of the study, preoperative US was performed by different ultrasonographers. To limit inter-observer variability, all measurements were taken by a single observer. The assessment of some criteria (e.g., cortical thickness) was sometimes challenging as it relied on retrospectively acquired still US images. Standardized prospective evaluation of the US-CKD score, which may also be useful in the follow-up of CKD alone, is required in a larger cohort and with multivariable analysis to confirm our results and draw causal inferences.

## 5 Conclusion

Evaluation of US findings consistent with CKD on the kidney contralateral to UO is beneficial to assess the postoperative evolution of SCr in cats treated unilaterally with an SUB device. In particular, the identification of severe signs of CKD in the contralateral kidney is associated with long-term IRIS stage IV with high specificity. However, measurements of renal parenchymal and hydronephrosis areas were not useful in predicting long-term renal recovery in cats after surgical decompression.

## Data Availability

The raw data supporting the conclusions of this article will be made available by the authors, without undue reservation.
