# Supplementary material for: Association of preoperative ultrasonographic parameters of the contralateral kidney with long-term serum creatinine in cats treated for unilateral ureteral obstruction
Source: Front Vet Sci. 2025 Jan 22;12:1518713. doi: 10.3389/fvets.2025.1518713 (PMC11795516; doi:10.3389/fvets.2025.1518713)
Supplement: Supplementary file 1 [file Table_1.DOCX]

Supplementary Material

**Figure S1**: Inclusion and exclusion criteria. SUB: subcutaneous ureteral bypass


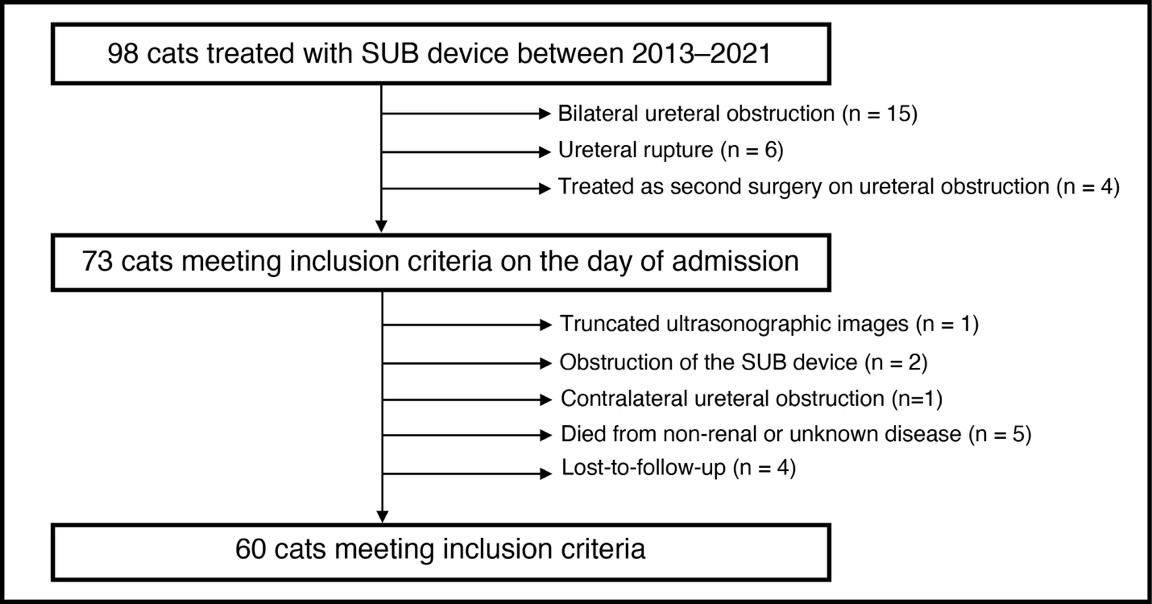


**Table S1**: Evaluation of the association between epidemio-clinical parameters and long-term serum creatinine in cats with unilateral ureteral obstruction. *Values are percentage or medians, values in brackets represent interquartile range.*

|  | Group A | Group B | *P* value |
| --- | --- | --- | --- |
| Age (years) | 6.2 [4.2 – 7.6] | 6.0 [4.5 – 8.9] | 0.43 |
| Weight (kg) | 3.6 [3 – 4.4] | 3.7 [3.4 – 4.5] | 0.30 |
| Duration of clinical signs (days) | 10.5 [7 – 23] | 10 [6 – 30] | 0.97 |
| Preoperative creatinine (mg/dL) | 5.2 [2.8 – 9.1] | 6.8 [5.6 – 8.6] | 0.28 |
| Purebred cat | 42% | 53% | 0.44 |
| Female | 72% | 42% | 0.03 |
| Neutered | 79% | 94% | 0.26 |
| Per-operative pelvic bacteriuria | 19% | 19% | 1 |
| Long-term bacteriuria | 24% | 18% | 1 |

**Table S2**: Evaluation of the association between ultrasonographic parameters of the obstructed kidney and long-term serum creatinine in included cats with unilateral ureteral obstruction. *Values are medians, values in brackets represent interquartile range.*

|  | Group A | Group B | *P* value |
| --- | --- | --- | --- |
| Total renal area (cm^2^) | 9.4 [7.7 – 11.6] | 9.2 [8.5 – 11.3] | 0.91 |
| Pelvic area (cm^2^) | 1.9 [1.4 – 3.1] | 2.6 [1.1 – 3.7] | 0.89 |
| RPA (cm^2^) | 7.7 [4.7 – 9.2] | 7.7 [6.6 – 8.7] | 0.82 |
| PHAR (cm^2^) | 4.1 [1.6 – 5.5] | 2.9 [2.4 – 6.1] | 0.94 |
| Renal length (cm) | 4.4 [3.9 – 4.7] | 4.3 [3.8 – 4.6] | 0.61 |
| Pelvic diameter in transverse plane (mm) | 6.9 [4.6 – 9.6] | 7.8 [6.3 – 13.4] | 0.12 |
| Pelvic diameter in sagittal plane (mm) | 9.7 [8.7 – 11.9] | 11.2 [8.3 – 14.9] | 0.39 |
| Renal cortical thickness (mm) | 4.4 [3.6 – 5.1] | 4.2 [3.7 – 4.8] | 0.65 |
| Renal parenchymal thickness (mm) | 8.4 [6.9 – 10.1] | 8.3 [7.7 – 10.2] | 0.81 |
| Cranial ureteral diameter (mm) | 4.9 [3.4 – 6.5] | 4.3 [3.2 – 6] | 0.36 |
| Ureteral diameter upstream obstruction site (mm) | 3.1 [2.1 – 3.8] | 2.9 [2.3 – 3.1] | 0.56 |
| US-CKD score | 3 [1 – 5] | 3 [2 – 5] | 0.56 |

*RPA = Renal Parenchymal Area; PHAR = Parenchyma to Hydronephrosis Area Ratio; US-CKD = ultrasonographic chronic kidney disease*
